# An analysis of secular trends in method-specific suicides in Japan, 1950–1975

**DOI:** 10.1186/s12963-017-0131-7

**Published:** 2017-04-05

**Authors:** Eiji Yoshioka, Yasuaki Saijo, Ichiro Kawachi

**Affiliations:** 1 Department of Social Medicine, Asahikawa Medical University, Midorigaoka-higashi 2-1-1-1, Asahikawa, Hokkaido Japan; 2 Department of Social and Behavioral Sciences, Harvard T.H. Chan School of Public Health, 677 Huntington Avenue, Kresge Building 7th Floor, Boston, MA USA

**Keywords:** Suicide, Epidemiology, Secular trends, Poisoning due to solid or liquid substances

## Abstract

**Background:**

In Japan, a dramatic rise in suicide rates was observed in the 1950s, especially among the younger population, and then the rate decreased rapidly again in the 1960s. The aim of this study was to assess secular trends in method-specific suicides by gender and age in Japan between 1950 and 1975. We paid special attention to suicides by poisoning (solid and liquid substances), and their contribution to dramatic swings in the overall suicide rate in Japan during the 1950s and 1960s.

**Methods:**

Mortality and population data were obtained from the Vital Statistics of Japan and Statistics Bureau, Ministry of Internal Affairs and Communications in Japan, respectively. We calculated method-specific age-standardized suicide rates by gender and age group (15–29, 30–49, or 50+ years).

**Results:**

The change in the suicide rate during the research period was larger in males than females in all age groups, and was more marked among people aged 15–29 years compared to those aged 30–49 years and 50 years or over. Poisoning by solid and liquid substances overwhelmingly contributed to the dramatic change in the overall suicide rates in males and females aged 15–49 years in the 1950s and 1960s. For the peak years of the rise in poisoning suicides, bromide was the most frequently used substance.

**Conclusions:**

Our results for the 1950s and 1960s in Japan illustrated how assessing secular trends in method-specific suicides by gender and age could provide a deeper understanding of the dramatic swings in overall suicide rate. Although rapid increases or decreases in suicide rates have been also observed in some countries or regions recently, trends in method-specific suicides have not been analyzed because of a lack of data on method-specific suicide in many countries. Our study illustrates how the collection and analysis of method-specific data can contribute to an understanding of dramatic shifts in national suicide rates.

## Background

Suicide is a significant yet preventable public health problem. In 2012, suicide accounted for 1.4% of all deaths worldwide, making it the 15^th^-leading cause of death [1]. Although the global estimate of the age-standardized suicide rate fell 26% (23% in men and 32% in women) during the 12-year period from 2000 to 2012 [1], very different patterns were seen by country or region. For example, large decreases in the age-standardized suicide rates from 2000 to 2012 were observed in China and Russia, while huge increases were observed in South Korea and Morocco [1]. However, the reason why the changes in suicide rates varied over the 12-year period has been unknown.

Although social, economic, cultural, and psychological factors are significant contributors to transitions of suicide rates, there is good evidence that the availability and popularity of lethal methods is also important [2, 3]. The increased use of pesticides in many agrarian societies during the second half of the 20th century, domestic coal gas in the 1920s and 1930s in the UK, and motor vehicles in the 1970s and 1980s in the UK were associated with an increase in suicides [4–6]. These findings indicate that analyzing trends in method-specific suicides may help to understand the background of the recent trends in overall suicides rates in some countries where rapid changes have recently been observed; however, many countries unfortunately do not collect data on the methods used in suicide [1]. Only 76 of the 194 WHO member states reported data on methods of suicide in the WHO mortality database between 2005 and 2011, and this means that the methods used in 72% of global suicides are unclear [1].

In Japan, a dramatic rise in a suicide rate was observed in the 1950s, and then the rate decreased just as rapidly in the 1960s [7, 8]. The rate in Japan was one of the highest worldwide in the 1950s [9]. It has been suggested that this change may be associated with the traumatic or difficult experiences of Japanese people during and after World War II and socioeconomic factors, such as unemployment rates, in the 1950s and 1960s in Japan [7, 8, 10, 11]. Concerning suicide methods used frequently during these periods in Japan, Araki et al. reported that suicide rates due to poisoning by solid and liquid substances in young people changed markedly in this period, and that these means were responsible for the rapid change [12]. However, little is known about how much impact the change in poisoning suicide rates during this period had on the overall suicide rate and what kind of solid and liquid substances were commonly used for suicide during this period.

In this research, we assessed secular trends in method-specific suicides by gender and age in Japan between 1950 and 1975. Specifically, we investigated the contribution of different methods of suicide to the overall suicide rate for males and females in Japan from 1950 to 1975 for three age groups (15–29, 30–49, and 50+) and compared the contribution of specific types of poisons to the overall suicide rate in these gender-age-specific cohorts when the national suicide rate was at its peak (1958–1960) to their contribution after the national suicide rate had dropped dramatically (1965–1967). By examining historical data we seek to illustrate how the analysis of trends in method-specific suicides can help to explain (and possibly prevent) the rapid changes that have recently been observed in other countries, e.g., South Korea and China [1].

## Methods

Gender-specific suicide mortality data for Japan between 1950 and 1975, by year in 5-year age bands, were obtained from the Vital Statistics of Japan [13]. Mid-year population estimates by gender and in 5-year age bands for Japan were provided by the Statistics Bureau, Ministry of Internal Affairs and Communications in Japan [14]. In this research, we divided age into three groups (15–29, 30–49, and 50+ years) and compared their trends. Data on suicide victims 14 years old or younger were not analyzed in this study. We calculated age-standardized suicide rates within each of these three age bands using the world population structure as a standard [15]. All suicide death rates in the figures were expressed as numbers of deaths per 100,000 men or women. Three-year moving averages that were centered on the last year of each 3-year period were then calculated to smooth the annual rates. And thus, although we obtained suicide mortality data between 1950 and 1975, the suicide rates based on the 3-year moving averages were restricted to the period from 1952 to 1975.

To identify the method of suicide, the underlying causes of death according to the ICD (International Classification of Diseases) codes were used (Table 1). We combined “Gases,” “Jumping,” “Cutting,” “Firearms,” etc. into the “Other and unspecified” category because the number of suicides using these methods was comparatively small during the research period. Moreover, we could obtain detailed information on what kind of solid or liquid substances were used for poisoning suicides in the years 1958–1967. The data between 1958 and 1967 were based on detailed items from the ICD-7 categories E970 (analgesic and soporific substances) and E971 (other solid and liquid substances) [13]. The E970 category consisted of four items: “morphine (morphine and other opium derivatives),” “barbiturate (barbiturate and its derivatives),” “bromide,” and “other analgesics (other and unspecified analgesic and soporific substances).” The E971 category also consisted of four items: “cyanide (cyanide compounds),” “pesticides (organic phosphorus preparations for agricultural chemicals),” “other phosphates (other phosphorus preparations)” and “other substances (other and unspecified solid and liquid substances).” For the remaining years, we could not obtain detailed information on solid or liquid substances which were used for poisoning suicides.

**Table 1:** ICD codes used for methods of suicide in Japan

	ICD-6	ICD-7	ICD-8
	1950–1957	1958–1967	1968–1975
Suicide	E970–E979	E970–E979	E950–E959
Poisoning other than gases	E970–E971	E970–E971	E950
Hanging	E974	E974	E953
Drowning	E975	E975	E954
Others and unspecified (including “gases,” “jumping,” “cutting,” “firearms,” etc.)	E972–E973, E976–E979	E972–E973, E976–E979	E951–E952, E955–E959

We compared the absolute and relative contributions, and examined the potential influence of demographic correlates (age and gender), for poisoning due to solid or liquid substances and other suicide methods to overall suicides between 1958–1960 and 1965–1967. The absolute contribution simply equals the net change of suicide rate between 1958–1960 and 1965–1967. On the other hand, the relative contribution equals the absolute contribution of a specified suicide method divided by the change in overall suicide rate over the period. This represents the proportion of the change in overall suicide rate that can be explained by that particular method of suicide during the period. We chose this period since we could obtain detailed data on suicide methods including specific substances used for poisoning suicides. The overall suicide rate in Japan reached its peak around the years 1958–1960, and then decreased sharply. Therefore, we think that examining the change in this period would clarify how each of the specified suicide methods played a role when the overall suicide rate decreased.

## Results

**Fig. 1:**
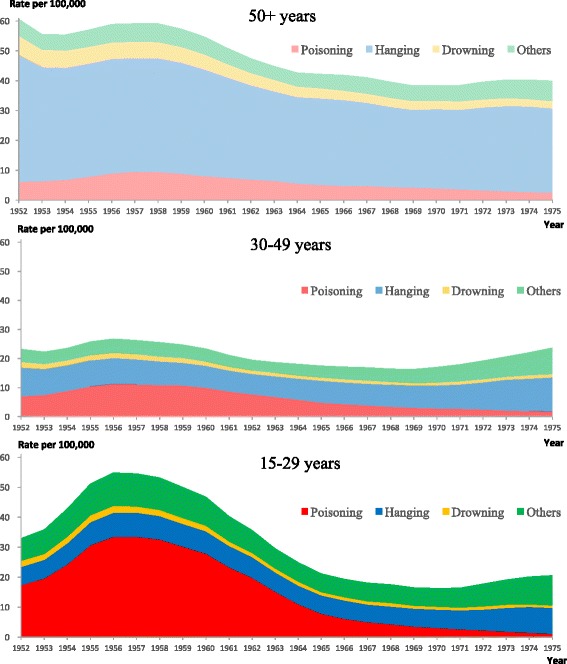
Secular trends in age-standardized suicide rates for males by age and method used in Japan, 1952–1975 (3-year moving averages)

**Fig. 2:**
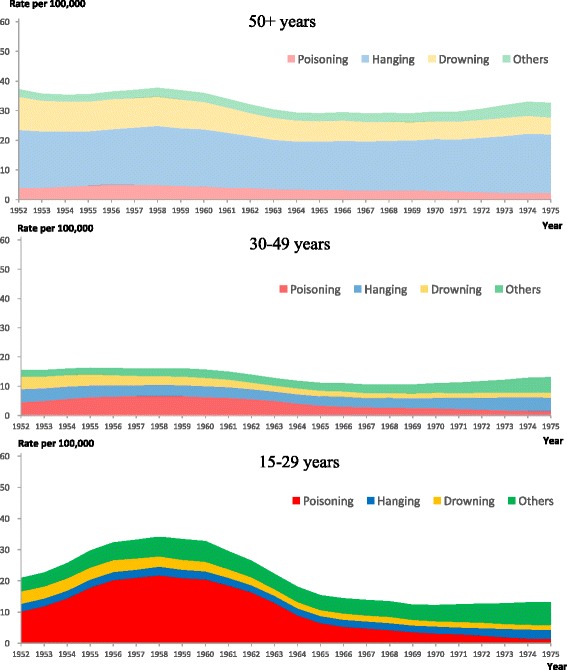
Secular trends in age-standardized suicide rates for females by age and method used in Japan, 1952–1975 (3-year moving averages)

Figures 1 and 2 show secular trends in age-standardized suicide rates by age and method used in Japan during the research period for males and females, respectively. For males and females aged 15–29 years, suicide rates for poisoning increased in the early 1950s, reached a peak in the late 1950s, and then decreased from the 1960s onward. In this age group, poisoning was the overwhelmingly dominant method in both genders from the early 1950s through the early 1960s. Dramatic changes in overall suicide rates were also observed in the 1950s and 1960s, and the overall suicide rates were almost parallel to the poisoning rates during this period. Although the overall (as well as) poisoning suicide rates of males were much higher than those of females, the secular trends showed similar tendencies between males and females in this age group. For males and females aged 30–49 and 50+ years, similar changes in poisoning suicide rates in the 1950s and 1960s were observed, although they were less marked than the trends among 15–29 year olds. Hanging was by far the most common method in those aged 50+ years during the research period.

**Fig. 3:**
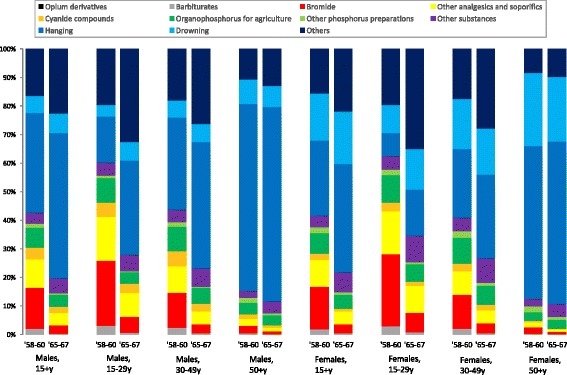
Distribution of suicide methods by gender and age in Japan in 1958–1960 and 1965–1967

Figure 3 show distribution of suicide methods by gender and age in the years 1958–1960 and 1965–1967. For males and females aged 15–29 years, proportions of poisoning in overall suicides decreased markedly from about 60% in 1958–1960 to about 30% in 1965–1967. For males and females aged 30–49 years, proportions of poisoning suicides also decreased from 40–43% in 1958–1960 to 23–27% in 1965–1967. For males and females aged 50+ years, comparatively small decreases in proportions of poisoning suicides were observed between 1958–1960 and 1965–1967. Tables 2, 3 and 4 shows the absolute and relative contribution of method-specific suicides to decreases in overall suicides between 1958–1960 and 1965–1967 for males and females aged 15–29, 30–49, and 50+ years. Regardless of gender and age, decreases in poisoning and non-poisoning suicide rates between 1958–1960 and 1965–1967 were observed. The decreases in poisoning suicide rates were much larger for males and females aged 15–29 years than those aged 30–49 and 50+ years. The share of the decrease in suicide rate accounted for by poisoning suicides during the period was much smaller in males and females aged 50+ years than those aged 15–29 and 30–49 years. For males and females aged 50 years or over, hanging was the greater contributor to the change in the overall suicide rate. Of the solid/liquid substances used as suicide methods, bromides were the most commonly used in 1958–1960 in both males and females aged 15–29 years, followed by other analgesics and pesticides. Bromides were also the most common poisoning substances in males and females aged 30–49 years, while organophosphates (used in agriculture) were the most common for those aged 50+ years. Suicide rates due to nearly all forms of poisoning decreased between 1958–1960 and 1965–1967.

**Table 2:** Age-standardized suicide rates (per 100,000 population) and changes in rates by method used between 1958–1960 and 1965–1967 for males and females aged 15–29 years, Japan

	Male				Female			
	Rate in 1958–1960	Rate in 1965–1967	Difference in rates (%) ^a^		Rate in 1958–1960	Rate in 1965–1967	Difference in rates (%) ^a^	
*Overall*	46.8	18.1	−28.7	(100)	32.7	13.8	−18.9	(100)
*Non-poisoning*	18.9	13.1	−5.8	(20.3)	12.3	9.1	−3.2	(17.0)
Hanging	7.5	5.9	−1.6	(5.6)	2.7	2.2	−0.4	(2.1)
Drowning	1.8	1.2	−0.7	(2.4)	3.2	2.0	−1.2	(6.3)
Others	9.6	6.0	−3.5	(12.3)	6.5	4.9	−1.6	(8.5)
*Poisoning*	27.9	5.0	−22.9	(79.7)	20.4	4.8	−15.7	(83.0)
Opium derivatives	0.01	0	−0.01	(0.04)	0.003	0	−0.003	(0.01)
Barbiturates	1.4	0.09	−1.3	(4.6)	0.9	0.1	−0.8	(4.5)
Bromide	10.5	1.0	−9.5	(33.1)	8.2	0.9	−7.3	(38.7)
Other analgesics and soporifics	7.2	1.5	−5.6	(19.6)	4.9	1.3	−3.6	(19.1)
Cyanide compounds	2.3	0.6	−1.7	(6.0)	1.0	0.2	−0.8	(4.3)
Organophosphartes for agriculture	4.0	0.7	−3.3	(11.4)	3.1	0.9	−2.3	(11.9)
Other phosphorus preparations	0.4	0.05	−0.4	(1.3)	0.6	0.1	−0.5	(2.9)
Other substances	2.1	1.0	−1.0	(3.5)	1.6	1.3	−0.3	(1.7)

^a^ “%” represents a percentage of the difference between the rate of a specified suicide method and that of overall suicides

**Table 3:** Age-standardized suicide rates (per 100,000 population) and changes in rates by method used between 1958–1960 and 1965–1967 for males and females aged 30–49 years, Japan

	Male				Female			
	Rate in 1958–1960	Rate in 1965–1967	Difference in rates (%) ^a^		Rate in 1958–1960	Rate in 1965–1967	Difference in rates (%) ^a^	
*Overall*	23.3	16.9	−6.4	(100)	15.7	10.6	−5.1	(100)
*Non-poisoning*	13.4	13.1	−0.3	(4.3)	9.4	7.8	−1.6	(30.8)
Hanging	7.5	7.5	0.0	(0.1)	3.8	3.1	−0.7	(13.7)
Drowning	1.4	1.1	−0.3	(5.2)	2.8	1.7	−1.0	(20.5)
Others	4.4	4.5	0.1	(−1.0)	2.8	2.9	0.2	(−3.3)
*Poisoning*	10.0	3.9	−6.1	(95.7)	6.3	2.8	−3.5	(69.2)
Opium derivatives	0.003	0	−0.003	(0.04)	0.005	0	−0.005	(0.1)
Barbiturates	0.5	0.1	−0.5	(7.4)	0.3	0.04	−0.3	(5.2)
Bromide	2.8	0.5	−2.2	(34.9)	1.8	0.4	−1.4	(28.4)
Other analgesics and soporifics	2.1	0.7	−1.3	(21.0)	1.3	0.5	−0.8	(15.7)
Cyanide compounds	1.2	0.4	−0.8	(12.2)	0.4	0.2	−0.2	(4.5)
Organophosphates for agriculture	2.0	0.9	−1.1	(16.8)	1.4	0.7	−0.7	(13.7)
Other phosphorus preparations	0.3	0.1	−0.3	(4.1)	0.4	0.1	−0.2	(4.9)
Other substances	1.0	1.1	0.1	(−0.8)	0.7	0.9	0.2	(−3.4)

^a^ “%” represents a percentage of the difference between rates of a specified suicide method and that of overall suicides

**Table 4:** Age-standardized suicide rates (per 100,000 population) and changes in rates by method used between 1958–1960 and 1965–1967 for males and females aged 50 years or above, Japan

	Male				Female			
	Rate in 1958–1960	Rate in 1965–1967	Difference in rates (%) ^a^		Rate in 1958–1960	Rate in 1965–1967	Difference in rates (%) ^a^	
*Overall*	54.8	41.1	−13.7	(100)	36.0	29.1	−6.9	(100)
*Non-poisoning*	46.7	36.4	−10.3	(75.4)	31.5	26.0	−5.5	(80.1)
Hanging	35.9	27.9	−8	(58.5)	19.3	16.5	−2.7	(39.8)
Drowning	4.8	3.0	−1.8	(12.9)	9.2	6.6	−2.6	(38.2)
Others	6.0	5.4	−0.6	(4.1)	3.0	2.8	−0.1	(2.1)
*Poisoning*	8.1	4.7	−3.4	(24.6)	4.5	3.1	−1.4	(19.9)
Opium derivatives	0.004	0	−0.004	(0.03)	0	0	0	(0)
Barbiturates	0.2	0.1	−0.2	(1.1)	0.1	0.04	−0.1	(0.9)
Bromide	1.4	0.4	−1.0	(7.5)	0.8	0.2	−0.6	(8.7)
Other analgesics and soporifics	1.2	0.5	−0.7	(5.0)	0.6	0.3	−0.3	(4.3)
Cyanide compounds	0.9	0.3	−0.5	(3.8)	0.2	0.04	−0.2	(2.2)
Organophosphates for agriculture	2.1	1.4	−0.7	(4.9)	1.1	0.9	−0.2	(2.9)
Other phosphorus preparations	0.9	0.3	−0.6	(4.8)	0.7	0.3	−0.4	(6.2)
Other substances	1.4	1.7	0.3	(−2.4)	0.9	1.3	0.4	(−5.5)

^a^ “%” represents a percentage of the difference between rates of a specified suicide method and that of overall suicides

## Discussion

### Main findings

In this research, we investigated the method-specific suicide trends by age and gender in Japan in the 1950s and 1960s. A rapid rise in suicide rate was observed in the 1950s, and then the rate decreased just as rapidly in the 1960s. The change in the suicide rate during this period was more marked among people aged 15–29 years compared to those aged 30–49 and 50 years or over. Poisoning by solid and liquid substances overwhelmingly contributed to the dramatic change in the overall suicide rates in males and females aged 15–49 years in the 1950s and 1960s. For the peak years of the rise in poisoning suicides, bromide was the most frequently used substance. The results of our research show that assessing secular trends in method-specific suicides by gender and age can contribute to a deeper understanding of the dramatic increase and decrease in the overall suicide rate in Japan in the 1950s and 1960s. Although rapid increases or decreases in suicide rates have been also observed in some countries or regions recently (e.g., South Korea, China), trends in method-specific suicides have not been analyzed because of a lack of data on method-specific suicide in many countries. Our study illustrates how the collection and analysis of method-specific data can contribute to an understanding of dramatic shifts in national suicide rates.

### Limitations

The study had several limitations that deserve discussion. First, although the data used in this analysis are based on data from official death certificates, completeness of suicide death recording may have changed over time as stigma associated with suicide could have changed. Moreover, changes occurred in the ICD classification system over the time period studied. However, visual inspection of the figures revealed no obvious discontinuities in the overall and method-specific suicide rates in the years when successive revisions of the ICD were implemented in Japan. We think that these changes could not fully explain the dramatic change in the suicide rates among young Japanese in the 1950s and 1960s. Second, we obtained data on the specific solid/liquid substances used in suicides for only a limited set of years. However, since we obtained data for the years between 1958 and 1967, we were able to observe a sharp decrease in the rate of poisoning suicides from the peak. Therefore, we clarified what kind of solid/liquid substances were the most frequently used in the peak years and how the mortality rate due to each of the substances changed. Last, the study was descriptive in design, and the delineation of the complex relationships among risk factors for suicide was beyond the scope of this study.

### Context of dramatic change in suicide rate in 1950s and 1960s in Japan

Motohashi examined the effects of socioeconomic factors on secular trends in suicide rates in Japan for the period 1953–1972 [10], and the result was that the suicide trends were almost parallel to those of unemployment rates in both men and women. However, if the socioeconomic factors in the 1950s and 1960s had influenced all method-specific suicides, all of the rates would have changed in a similar way. Actually, the changes in all method-specific suicide rates other than solid and liquid poisoning were less marked during this period. Thus, we think that socioeconomic factors do not fully explain why the drastic changes in overall suicides were observed in the 1950s and 1960s.

Bromide was the most frequently used substance of all liquids/solids among males and females aged 15–29 and 30–49 years in the peak years of the poisoning suicide rates. In Japan, bromide, the product name of which was “Calmotin,” had been available as an over-the-counter medicine by the early 1930s at the latest, and was commonly used as a hypnotic and sedative [16, 17]. Thus, easy access to this drug could lead to a large number of suicides due to this drug. Other analgesics were the second-most frequently used substance category among males aged 15–29 and 30–49 years and females aged 15–29 years in the late 1950s. Meprobamate was a carbamate derivative used as an anxiolytic drug and included in the category “other analgesics.” This drug was sold as an over-the-counter medicine in Japan starting in 1957 [18]. It was suggested that drug companies’ advertisements and mass media reporting could lead to an increase in users of the drug and suicides due to the drug [18]. This social circumstance, combined with the socioeconomic crisis after the war, might have led to the dramatic increase in poisoning suicides in the 1950s.

The Pharmaceutical Affairs Law (Act No. 145 of 1960), which was a law regulating the manufacturing, importation, and sale of drugs and medical devices in Japan, went into effect in February 1961 [19]. This law classified most hypnotics and sedatives, including bromisoval (Calmotin) and meprobamate, as habit-forming drugs and regulated the sale of these drugs. In addition, the Agricultural Chemicals Control Act (Act No. 82 of 1948) and Poisonous and Deleterious Substances Control Act (Act No. 303 of 1950) were promulgated in July 1948 and December 1950, respectively. These acts were established for the purpose of strengthening regulations on the production, import, sale, and handling of pesticides, cyanide, or other poisonous substances. These regulations may have been effective in the prevention of misuses of these substances only after the mid-1950’s and thus may have contributed to a decrease in suicides by these substances.

The changes in suicide rates due to poisoning by solid and liquid substances during the 1950s and 1960s were much more marked for younger Japanese than for older groups. One explanation for this age difference is that youth are more likely to imitate societal trends and to attempt copycat suicides. For example, there was a major rise in hanging suicides in young Australian men and women in the 1980s [2].

### Implications for suicide prevention

Choice of suicide methods is not likely to be random, but rather influenced by a complex constellation of psychosocial, environmental, and biological factors which precede the decision to commit suicide [2]. Physical availability and sociocultural acceptability are viewed as two key factors that influence the choice of suicide method [20]. Physical availability refers to the extent to which a particular method of suicide is accessible to the individual, while sociocultural acceptability is a measure of the extent to which a person’s choice of method is shaped and circumscribed by the norms, traditions, and moral attitudes of their culture. There is good evidence that changing the physical availability of lethal methods is an important contributor to suicide rates [2, 3]. Previous research has shown that method-specific suicide rates decreased after firearm control legislation, restrictions on pesticides, and detoxification of domestic gas [5, 21–23]. In countries where a particular method is common, restriction of that means could also lead to lower overall suicide rates [3, 24]. Means restriction is most effective when the method is common and highly lethal [3]. Although substitution of one method with another does happen, people who attempt suicide with less lethal means have an increased chance of survival when reduced access to a highly lethal method is possible. The present study revealed that in Japan during the 1950s and 1960s, the legal restrictions on access to medical drugs and poisonous substances resulted in dramatic falls in suicide rates, especially among the young population, and confirmed that modification of the environment to restrict access to lethal means was a key element of suicide prevention efforts [1, 3, 24]. Since devising appropriate restriction policies requires a detailed understanding of the methods of suicide used in the community (as well as of the method preferred by different demographic groups within the community) [1], detailed research on the descriptive epidemiology of suicide will continue to be relevant.

## Conclusions

Overall suicide rates increased dramatically in young Japanese men and women in the early 1950s, reached a peak in the late 1950s, and then decreased sharply again in the 1960s. Poisoning by solid/liquid substances overwhelmingly contributed to the dramatic change in the overall suicide rates. Bromide was used in about 40% of the poisoning suicides among younger people in the years 1958–1960, representing the peak of the poisoning epidemic. Our results for the 1950s and 1960s illustrate how assessing secular trends in method-specific suicides by gender and age can provide a deeper understanding of the dramatic swings in overall suicide rate. Although rapid increases or decreases in suicide rates have been also observed in some countries or regions recently (e.g., South Korea, China), trends in method-specific suicides have not been analyzed because of a lack of data on method-specific suicide in many countries. Our study illustrates how the collection and analysis of method-specific data can contribute to an understanding of dramatic shifts in national suicide rates.

## Abbreviation

ICD: International Classification of Diseases
